# Competing opinion diffusion on social networks

**DOI:** 10.1098/rsos.171160

**Published:** 2017-11-01

**Authors:** Haibo Hu

**Affiliations:** Department of Management Science and Engineering, East China University of Science and Technology, Shanghai, People’s Republic of China

**Keywords:** social networks, complex networks, opinion diffusion, competition

## Abstract

Opinion competition is a common phenomenon in real life, such as with opinions on controversial issues or political candidates; however, modelling this competition remains largely unexplored. To bridge this gap, we propose a model of competing opinion diffusion on social networks taking into account degree-dependent fitness or persuasiveness. We study the combined influence of social networks, individual fitnesses and attributes, as well as mass media on people’s opinions, and find that both social networks and mass media act as amplifiers in opinion diffusion, the amplifying effect of which can be quantitatively characterized. We analytically obtain the probability that each opinion will ultimately pervade the whole society when there are no committed people in networks, and the final proportion of each opinion at the steady state when there are committed people in networks. The results of numerical simulations show good agreement with those obtained through an analytical approach. This study provides insight into the collective influence of individual attributes, local social networks and global media on opinion diffusion, and contributes to a comprehensive understanding of competing diffusion behaviours in the real world.

## 1. Introduction

In real life, we usually have specific viewpoints on certain topics, such as consumer products, life styles, celebrities, etc. The opinions are generally formed based on certain private information or personal life experience. By communicating with friends, family and colleagues, our opinions can be influenced and changed by social networks to which we are exposed. With the rise of social networking sites like Facebook and social media like Twitter, social networks have penetrated our lives from multiple dimensions, and opinion diffusion and evolution, or more broadly, the evolution of social dynamics on networks has also attracted the attention of researchers from diverse disciplines [1–3].

In recent years, opinion diffusion on networks has been intensely investigated from many different perspectives using the approaches of network science and statistical physics. An individual opinion can be defined by a finite number of integers, and such discrete-opinion models include voter model [4–6], Axelrod model [7], Sznajd model [8], majority rule models [9–11] and the model based on social impact theory [12]. The Axelrod model can be viewed as multiple coupled voter models because it features the main ingredients of a voter model [13]. In these models, people are influenced by their neighbours and update their opinions, often according to some version of a majority rule or imitation. Opinions can also be represented by real numbers, and such continuous-opinion models include the DeGroot model [14,15], Friedkin model [16,17], Deffuant model [18] and Hegselmann–Krause model [19]. The latter two models involve bounded confidence between people [20], which means that if the opinions of two people are too far apart, they do not influence each other. These two models typically result into a clusterization of opinions [21,22].

The dynamical process of opinion diffusion can have a natural absorbing state or consensus at the steady state, in which all people share the same opinion. However, coexistence of diverse opinions is also possible. Opinion or product diffusion on networks has also been extensively studied in economics literature in which dynamic behaviours are commonly termed social learning [23]. For example, essentially the voter model and the DeGroot model are non-Bayesian models of social learning [24].

In fact, opinions not only diffuse on social networks, but they also compete for share when the opinions are related to certain interests, for instance opinions on controversial issues or consumer products. The supporters of some opinions or fans of some brands will try to influence or persuade other people with different opinions or different brand preferences. Recently, theoretical models have been proposed to study competing memes, products or viruses spreading on networks. For example, Prakash *et al.* studied a competing diffusion model, Susceptible–Infected_1_–Infected_2_–Susceptible (SI_1_I_2_S), based on the traditional virus propagation model [25]. They found that under realistic conditions, the stronger virus will completely wipe out the weaker one, which explains the ‘winner takes all’ phenomenon in the real world, such as Facebook versus Myspace and Blu-ray versus HD-DVD. The model assumes that there is perfect competition; however, this is not always true, for example, a user could use both Firefox and Google Chrome as browsers. For this situation, Beutel *et al.* proposed a model, Susceptible–Infected_1 *or* 2_–Susceptible (SI_1|2_S), and studied the scenarios between full competition and no competition. They found that there is a phase transition: if the competition is harsher than a critical level, then ‘winner takes all’; otherwise, the weaker virus survives [26]. Based on classical compartmental models, Ribeiro and Faloutsos also proposed a competitive model which mimics website popularity competition and explains the rise of Facebook and the decline of Myspace [27]. Some other competitive diffusion models based on a branching process [28], Susceptible–Infected–Susceptible (SIS) [29] or Susceptible–Infected–Recovered (SIR) [30] epidemic spreading model have also been studied.

Competitive diffusion processes not only occur on a single network, but also operate on multilayer networks [31,32], for example simultaneously on Twitter, Facebook and offline social networks for which each network constitutes a network layer. Usually these layers are not mutually independent, and they are connected through common people. Some people have accounts of multiple websites; they are called coupling nodes of multilayer networks and can influence people on different layers or spread information on some layer to another layer. In recent years, researchers have also put forward competitive memes or virus propagation models based on the SIS epidemic model on multilayer networks [33], such as the SI_1_I_2_S [34,35] and SI_1_SI_2_S models [36].

In the real-world scenarios, for instance in Twitter, multiple tweets spread through the network simultaneously, and during this process they interact and compete for users’ attention. Empirical research found that the competing diffusion decreases each other’s probability of spreading [37]. Recently, the wide adoption of social media has increased the competition among ideas for our finite attention. Weng *et al.* employed an agent-based model to study the competitive diffusion process, and the predictions of the model are consistent with empirical data from Twitter [38]. The competition for public attention on multiple topics promoted by various opinion leaders on social media has also attracted the attention of the field of computer visualization, and visual analysis approaches, such as timeline visualization [39,40], have also been proposed to study topic competition and cooperation on social media.

Most competitive diffusion models study the interaction of different memes or viruses; however, memes of diffusion are different from opinion dynamics. For the former, initially there are people who do not know the memes while, for the latter, each person has an initial opinion. In memes of diffusion, memes are propagated from the people who know them to those who do not know them, while in opinion diffusion initially there exist several different opinions distributed in the networks and the opinions interact with each other. Different from meme or information diffusion, it is difficult to obtain empirical data of opinion competition in social networks. Research on competing opinion diffusion by theoretical models is also largely missing. To fill this gap, in this paper, we will use an agent-based model derived from the voter model, a well-known opinion dynamics model, to investigate competing diffusion in social networks.

In the original voter model, each person is endowed with a binary opinion. The elementary step consists in choosing a first person and one of her/his nearest neighbours, both randomly. Then the first person sets her/his new opinion to be the same as the neighbour [6]. Besides modelling opinion evolution, the voter model is also valuable as a general framework for research on diffusion of products, innovations or consumption decisions, and despite its simplicity, its ability to model and predict real opinion diffusion and product adoption on both the individual and group levels has been validated by empirical data [41–43]. In this paper, we will make a few extensions to the original voter model.

The main contribution of the paper is defining a model of competing opinion diffusion within social networks to quantitatively evaluate the impact of various factors on people’s opinions. The proposed model is generic and can be applied to different scenarios in the real world. We will study that under the interaction of network topology, individual attributes and external influence, which opinion will ultimately pervade the entire social networks, under what conditions the different opinions will coexist, and what is the proportion of each opinion when coexisting.

## 2. Model

The previous research on the voter model usually focuses on binary opinions, supposes that each person has the same persuasiveness, and studies the endogenous influence of social networks on opinion evolution [6,44]. However, besides binary opinions, in the real world there exist many situations in which the number of available opinions is more than two. Owing to the differences in social, economic and cultural capital, different people may have different persuasiveness [45–47]. Mass media, such as television, radio, Internet, and print, can also influence people’s opinions [48,49]. To study competing opinion diffusion on networks, we make four extensions to the original voter model. The binary opinions are extended to multiple opinions, and committed or stubborn people who are usually opinion leaders or loyal fans in the real world are introduced into the model. In addition to social networks, the influence of mass media on people’s opinions is also considered. Also, each person is endowed with a value which indicates her/his persuasiveness or fitness.

Let *G*=(*V*,*E*) be a finite, undirected, unweighted and connected graph, where *V* is the set of vertices and *E* is the set of edges. We assume that the graph is simple, i.e. no vertex is connected to itself and there are no parallel edges. Many studies have found a positive association between a person’s degree and that person’s goal achievement, including creativity, job attainment, professional advancement, political influence and prestige [50,51]. Thus, a person’s degree can be a stand-in for her/his true fitness. Let *N* be the number of vertices and *f*_*k*_>0 be the fitness of people with degree *k*. We suppose that the total number of different opinions is *I*≥2, and the opinion which a person can take is a discrete value *i*=1,2,…,*I*. These different opinions are equivalent and mutually exclusive. Initially each person is randomly assigned an opinion. The basic update procedure is described as follows. At every time step, we choose randomly a person who will update her/his opinion, then we choose one of the person’s nearest neighbours with the probability proportional to the neighbour’s fitness, and finally we set the person’s new opinion to be the same as the neighbour. This step is repeated until the dynamic process reaches the steady state.

The proposed model is different from linear threshold and independent cascade models [52,53] although in these three models essentially people change their opinions or states due to the influence of their neighbours. In linear threshold or independent cascade model, each person can be in an active state or inactive state. For linear threshold model, an inactive person will become active if the fraction of her/his neighbours in an active state or the sum of the weights of the edges with an active neighbours exceeds her/his threshold. However, there are no thresholds in our model. In the independent cascade model, any active person has only one chance to activate her/his inactive neighbours; whether successful or not, the person will not influence her/his neighbours in the subsequent steps. By contrast, our model has no restrictions on the number of activations. In linear threshold and independent cascade models, all people in an active state will stay unchanged, but in our model except committed people, the states of all others will be affected by their neighbours.

Our model supposes that a person’s fitness relates to her/his degree. There are several existing works on the effects of degree-based social power on opinion evolution [54] and opinion formation by informed people or leaders [55,56] which provide insight into the influence of important people on opinion diffusion, although these works are based on bounded confidence model. For example, the model proposed by Jalili introduces leaders whose opinions are kept unchanged and centrality-based social power which are also included in our model [56].

## 3. Social influence

We first consider the influence of social networks on people’s opinions, which is usually termed social influence in social psychology. Assume that the degree distribution of the social network studied is *p*_*k*_, the fraction of people with opinion *i* in all people with degree *k* is *q*_*i*,*k*_, and the conditional probability that a person of degree *k* is connected to a person of degree *k*′ is *P*(*k*′ | *k*). At a time step, the probability that a degree-*k* person changes her/his opinion from not *i* to *i* is

3.1 $$p_{\bar{i}\to i}(k)=p_{k}(1-q_{i,k})\frac{\sum_{k^{\prime }}P(k^{\prime }\;|\;k)q_{i,k^{\prime }}f_{k^{\prime }}}{\sum_{j}\sum_{k^{\prime }}P(k^{\prime }\;|\;k)q_{j,k^{\prime }}f_{k^{\prime }}}.$$

Similarly, the probability that a degree-*k* person changes her/his opinion from *i* to not *i* is

3.2 $$p_{i\to \bar{i}}(k)=p_{k}q_{i,k}\left[1-\frac{\sum_{k^{\prime }}P(k^{\prime }\;|\;k)q_{i,k^{\prime }}f_{k^{\prime }}}{\sum_{j}\sum_{k^{\prime }}P(k^{\prime }\;|\;k)q_{j,k^{\prime }}f_{k^{\prime }}}\right].$$

Let 〈*k*〉 be the average degree of the network, *n*_*k*_=*Np*_*k*_ be the number of people with degree *k*, and *m*_*i*,*k*_=*n*_*k*_*q*_*i*,*k*_ be the number of people with degree *k* and opinion *i*. To obtain closed analytical solutions, we assume that the social network is degree uncorrelated. In this case $P(k^{\prime }\;|\;k)=k^{\prime }p_{k^{\prime }}/\sum_{k^{\prime }}k^{\prime }p_{k^{\prime }}=k^{\prime }p_{k^{\prime }}/\left\langle k\right\rangle$. Thus, equations (3.1) and (3.2) can be rewritten as

3.3 $$\begin{array}{rl} p_{\bar{i}\to i}(k) & =p_{k}(1-q_{i,k})\frac{\sum_{k^{\prime }}k^{\prime }f_{k^{\prime }}m_{i,k^{\prime }}}{\sum_{j}\sum_{k^{\prime }}k^{\prime }f_{k^{\prime }}m_{j,k^{\prime }}}\\ \;\text{and}\;p_{i\to \bar{i}}(k) & =p_{k}q_{i,k}\left(1-\frac{\sum_{k^{\prime }}k^{\prime }f_{k^{\prime }}m_{i,k^{\prime }}}{\sum_{j}\sum_{k^{\prime }}k^{\prime }f_{k^{\prime }}m_{j,k^{\prime }}}\right). \end{array}\}$$

We define

3.4 $$q_{i}^{f}=\frac{\sum_{k^{\prime }}k^{\prime }f_{k^{\prime }}m_{i,k^{\prime }}}{\sum_{j}\sum_{k^{\prime }}k^{\prime }f_{k^{\prime }}m_{j,k^{\prime }}}=\frac{\sum_{k^{\prime }}k^{\prime }f_{k^{\prime }}m_{i,k^{\prime }}}{\sum_{k^{\prime }}k^{\prime }f_{k^{\prime }}n_{k^{\prime }}}=\frac{\sum_{k^{\prime }}k^{\prime }f_{k^{\prime }}p_{k^{\prime }}q_{i,k^{\prime }}}{\sum_{k^{\prime }}k^{\prime }f_{k^{\prime }}p_{k^{\prime }}}$$

as the normalized weighted fraction of opinion *i*, and it is the fraction of the sum of product of degree and fitness for people with opinion *i*. Thus, equation (3.3) can be rewritten more simply as

3.5 $$\begin{array}{rl} p_{\bar{i}\to i}(k) & =p_{k}(1-q_{i,k})q_{i}^{f}\\ \;\text{and}\;p_{i\to \bar{i}}(k) & =p_{k}q_{i,k}(1-q_{i}^{f}). \end{array}\}$$

The evolution equation of *q*_*i*,*k*_ is [57]

3.6 $$\begin{array}{rl} dq_{i,k} & =\left[\frac{\left(p_{\bar{i}\to i}(k)-p_{i\to \bar{i}}(k)\right)}{p_{k}}\right]dt\\ & =(q_{i}^{f}-q_{i,k})\;dt. \end{array}$$

At the steady state d*q*_*i*,*k*_/d*t*=0, thus $\lim_{t\to \infty }q_{i,k}(t)=q_{i}^{f}$. Let $q_{i}=\sum_{k}n_{k}q_{i,k}/N$ be the fraction of people with opinion *i*, thus $\lim_{t\to \infty }q_{i}(t)=q_{i}^{f}$. In this case, finally there will be only one opinion in the network, which means that all people’s opinions will reach a consensus. From equation (3.6) we note that $q_{i}^{f}$ is a conserved quantity and its mean is a constant [6,57]. $q_{i}^{f}$ is also termed fixation probability [58,59] in biology or exit probability in physics [6], which is the probability that opinion *i* will finally occupy the whole network. Therefore, according to the model, the people with large degrees and high persuasiveness or fitness will be more influential and competitive in the opinion diffusion process. The preponderance of some opinion will be suppressed by the opinions with large *kf*_*k*_.

When all people have the same fitness, from equation (3.4) we obtain

3.7 $$q_{i}^{f}=\frac{\sum_{k^{\prime }}k^{\prime }p_{k^{\prime }}q_{i,k^{\prime }}}{\sum_{k^{\prime }}k^{\prime }p_{k^{\prime }}}=\frac{\sum_{k^{\prime }}k^{\prime }m_{i,k^{\prime }}}{N\langle k\rangle },$$

which is the fraction of the total degree of the people with opinion *i* in the total degree of the whole network. The characteristics of the voter model itself make it advantageous for nodes with large degrees. To suppress the advantages of these nodes, *f*_*k*_ should be a decreasing function of *k*. For instance, when *f*_*k*_∝1/*k*, we obtain $q_{i}^{f}=q_{i}$ and degree values no longer play a role.

**Figure 1.**
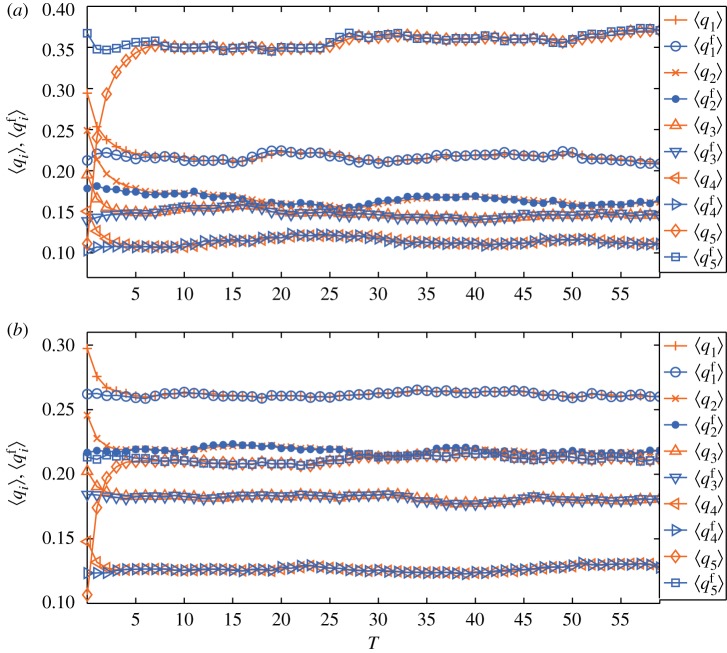
Evolutions of 〈*q*_*i*_〉 and $\left\langle q_{i}^{f}\right\rangle$. The unit of time *T* is *N* steps. (*a*) *f*_*k*_∝*k*^0.5^; (*b*) *f*_*k*_ follows uniform distribution in (0, 1). For both cases, $\left\langle q_{i}^{f}\right\rangle$ is a constant, and as time goes on, 〈*q*_*i*_〉 gradually approaches $\left\langle q_{i}^{f}\right\rangle$.

We perform numerical simulations on a Barabási–Albert (BA) network [60] of *N*=10^4^ and 〈*k*〉=6 to validate the analytical conclusions. Assume that initially the total number of different opinions is *I*=5, and the opinions are randomly assigned. We can obtain the mean values of *q*_*i*_ and $q_{i}^{f}$ by averaging 100 independent realizations starting from the same initial condition. To distinguish $q_{i}^{f}$ from *q*_*i*_, we assume that the 100 largest degree people hold opinion 5. First, we assume that *f*_*k*_∝*k*^0.5^ and the simulation results are shown in figure 1*a*. The large-degree people and their high fitness make initial $q_{5}^{f}$ much larger than *q*_5_. As predicted, $q_{i}^{f}$ is a conserved quantity, and as time goes on, the mean values of *q*_*i*_ approach mean values of $q_{i}^{f}$. Then we suppose that *f*_*k*_ follows a uniform distribution in (0,1) and the simulation results are shown in figure 1*b* which also validates the analytical conclusions.

According to equation (3.4), when *f*_*k*_∝*k*^0.5^, even though initially *q*_5_ is the smallest among *q*_*i*_, *q*^f^_5_ is the largest among $q_{i}^{f}$ due to the influence of large-degree people, which means that opinion 5 has the largest probability to pervade the whole network. By contrast, when *f*_*k*_ follows a uniform distribution, the people with large degrees lose their advantage. Even though initially *q*^f^_5_ is still much larger than *q*_5_, however, opinion 1 replaces 5 and becomes the most likely to occupy the network.

In this paper, we focus on the spread of opinions on unweighted networks. However, we will demonstrate that under certain conditions, opinion diffusion on weighted networks is a special case of our model. On weighted networks, we still choose randomly the first person *i*, but choose one of *i*’s neighbours *j* with probability proportional to the weight *w*_*ij*_ of the edge connecting *i* and *j*. Let *k*_*i*_ and *k*_*j*_ be the degrees of *i* and *j*, respectively, and note that empirical studies have found that approximatively the mean weight 〈*w*_*ij*_〉∝(*k*_*i*_*k*_*j*_)^*θ*^, where *θ* is a small positive value and usually *θ*<0.5 [61]. To obtain closed analytical results we can neglect the fluctuations and assume that *w*_*ij*_∝(*k*_*i*_*k*_*j*_)^*θ*^. In this case, we can obtain

3.8 $$q_{i}^{f}=\frac{\sum_{k^{\prime }}k^{\prime \theta +1}p_{k^{\prime }}q_{i,k^{\prime }}}{\sum_{k^{\prime }}k^{\prime \theta +1}p_{k^{\prime }}}.$$

Thus, the conclusion for weighted networks is a special case when *f*_*k*_∝*k*^*θ*^. It is noteworthy that, for large *θ*, theoretical prediction by the mean-field approach will produce greater error [44]. Further on weighted networks, if we choose one of *i*’s neighbours *j* with probability proportional to *w*_*ij*_*f*_*k*_*j*__, $q_{i}^{f}$ will become

3.9 $$q_{i}^{f}=\frac{\sum_{k^{\prime }}k^{\prime \theta +1}f_{k^{\prime }}p_{k^{\prime }}q_{i,k^{\prime }}}{\sum_{k^{\prime }}k^{\prime \theta +1}f_{k^{\prime }}p_{k^{\prime }}}.$$

Next, we introduce committed or stubborn people in the model [57,62–65], which can represent opinion leaders in a social context. The committed people always stick to their original opinions and do not change their opinions over time. They can influence their friends; however, their friends never influence the committed people’s opinions. Empirical study has revealed that some people indeed show committed behaviour [66]. In social networks, we let a fraction of the people be committed ones and let the other people be non-committed or regular ones. Assume that the fraction of committed people with opinion *i* in all degree-*k* people is *s*_*i*,*k*_; in this case, equation (3.5) becomes

3.10 $$\begin{array}{rl} p_{\bar{i}\to i}(k) & =p_{k}(1-q_{i,k}-\sum_{j\neq i}s_{j,k})q_{i}^{f}\\ \;\text{and}\;p_{i\to \bar{i}}(k) & =p_{k}(q_{i,k}-s_{i,k})(1-q_{i}^{f}). \end{array}\}$$

Let *s* be the fraction of committed people and *s*_*i*_ be the fraction of committed people with opinion *i*. According to equation (3.6),

3.11 $$dq_{i,k}=\left[\left(1-\sum_{j}s_{j,k}\right)q_{i}^{f}-q_{i,k}+s_{i,k}\right]dt.$$

At the steady state

3.12 $$\lim_{t\to \infty }q_{i,k}(t)=\left(1-\sum_{j}s_{j,k}\right)q_{i}^{f}+s_{i,k},$$

thus,

3.13 $$\lim_{t\to \infty }q_{i}(t)=(1-s)q_{i}^{f}+s_{i}.$$

According to equations (3.4) and (3.11), we obtain

3.14 $$\frac{dq_{i}^{f}}{dt}=s_{i}^{f}-q_{i}^{f}\sum_{j}s_{j}^{f}.$$

At the steady state,

3.15 $$\lim_{t\to \infty }q_{i}^{f}(t)=\frac{s_{i}^{f}}{\sum_{j}s_{j}^{f}}.$$

According to equations (3.13) and (3.15), we obtain

3.16 $$\lim_{t\to \infty }q_{i}(t)=s_{i}+(1-s)\frac{s_{i}^{f}}{\sum_{j}s_{j}^{f}}.$$

It is obvious that only the opinions that committed people hold will survive. Equation (3.16) is a general conclusion. The final people holding opinion *i* are composed of two parts, one is from the committed ones and the other is from the people who are induced by the committed ones by social influence. In the model, social networks amplify the opinions of committed people and, from the right-hand side of equation (3.16), we find that the amplification factor is just $s_{i}^{f}/\sum_{j}s_{j}^{f}$.

If all committed people stick to opinion *i*, all non-*i* opinions will die out. Therefore, in the context of our model, to ensure that some opinion has the greatest influence in the whole society, there must be a lot of faithful people who have large degree values and have a strong persuasiveness that can affect many others.

**Figure 2.**
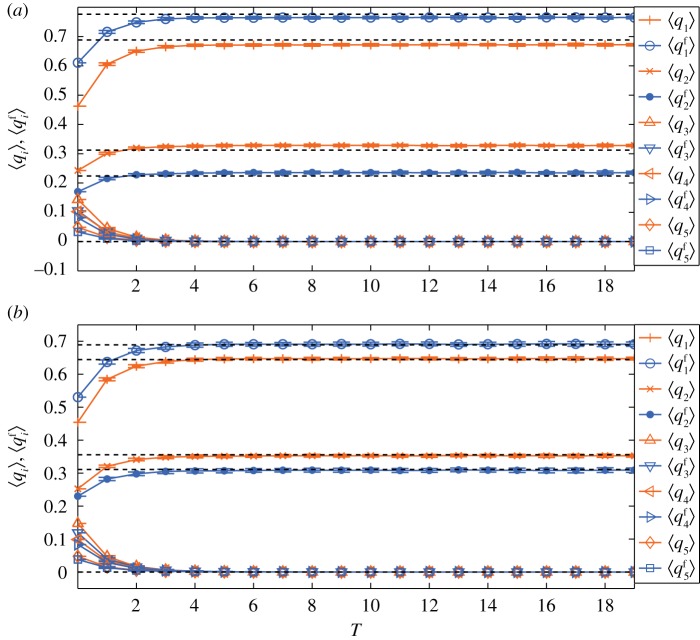
Evolutions of 〈*q*_*i*_〉 and $\left\langle q_{i}^{f}\right\rangle$. The unit of time *T* is *N*/(1−*s*) steps. The dashed lines indicate $\lim_{t\to \infty }q_{i}^{f}(t)$ and $\lim_{t\to \infty }q_{i}(t)$ obtained from equations (3.15) and (3.16). Error bars with ±1 standard deviation are also shown. (*a*) *f*_*k*_∝*k*^0.5^; (*b*) *f*_*k*_ follows a uniform distribution in (0, 1). For both cases, at steady state both 〈*q*_*i*_〉 and $\left\langle q_{i}^{f}\right\rangle$ are in good agreement with theoretical predictions.

Similarly, we perform numerical simulations on the BA network of *N*=10^4^ and 〈*k*〉=6. We suppose that initially the people, both with opinions 1 and 2, are a mix of committed and regular ones, while all the other people holding opinions 3, 4 and 5 are regular ones. In networks, a large number of committed people with large degrees will accelerate the convergence of the dynamic process. Thus, we assume that the 100 largest degree people are the committed ones with opinion 1, *s*_1_=0.3, *s*_2_=0.2 and *s*=0.5. The corresponding numerical simulation results are shown in figure 2, which are in good agreement with the analytical predictions. We note that at steady state, compared with figure 2*b*, in figure 2*a* *q*^f^_1_ is much larger than *q*_1_ due to the impact of *f*_*k*_.

## 4. Media effect

In the real world, apart from social networks, people are also influenced by mass media. A mass media outlet is a global source of information, and individual opinions can be influenced by the information they obtain via both local social networks and global media outlets [67]. We define a weight parameter *P*∈[0,1] that measures the relative intensity of the mass media with respect to the local social network. Thus, in the model, the individual opinion update rule can be revised as follows. At each time step, we choose randomly a person. If the person is committed, nothing happens; if the person is regular, her/his opinion will be affected by both mass media and social networks. When a regular person updates her/his opinion, she/he chooses the media opinion as her/his new opinion with probability *P*, and selects one of her/his friends and sets her/his new opinion to be the same as that of a friend’s with probability 1−*P*. The probability of choosing a friend is still proportional to the friend’s fitness. We assume that the media opinion is *m* (1≤*m*≤*I*). In this case, when *i*≠*m*, we obtain

4.1 $$\begin{array}{rl} p_{\bar{i}\to i}(k) & =p_{k}\left(1-q_{i,k}-\sum_{j\neq i}s_{j,k}\right)(1-P)q_{i}^{f}\\ \;\text{and}\;p_{i\to \bar{i}}(k) & =p_{k}(q_{i,k}-s_{i,k})[(1-P)(1-q_{i}^{f})+P]. \end{array}\}$$

Applying the preceding approach, we obtain

4.2 $$\lim_{t\to \infty }q_{i}^{f}(t)=\frac{s_{i}^{f}}{P+(1-P)\sum_{j}s_{j}^{f}}$$

and

4.3 $$\lim_{t\to \infty }q_{i}(t)=s_{i}+(1-s)(1-P)\frac{s_{i}^{f}}{P+(1-P)\sum_{j}s_{j}^{f}}.$$

When *P*=0, equations (4.2) and (4.3) are reduced to equations (3.15) and (3.16), respectively. Social networks amplify the influence of committed people with an amplification factor related to $\lim_{t\to \infty }q_{i}^{f}(t)$.

When *i*=*m*, similarly we can obtain

4.4 $$\begin{array}{rl} p_{\bar{i}\to i}(k) & =p_{k}\left(1-q_{i,k}-\sum_{j\neq i}s_{j,k}\right)[(1-P)q_{i}^{f}+P]\\ \;\text{and}\;p_{i\to \bar{i}}(k) & =p_{k}(q_{i,k}-s_{i,k})(1-P)(1-q_{i}^{f}) \end{array}\}$$

and finally,

4.5 $$\lim_{t\to \infty }q_{i}^{f}(t)=\frac{s_{i}^{f}+P(1-\sum_{j}s_{j}^{f})}{P+(1-P)\sum_{j}s_{j}^{f}}$$

and

4.6 $$\begin{array}{rl} \lim_{t\to \infty }q_{i}(t) & =s_{i}+(1-s)(1-P)\frac{s_{i}^{f}}{P+(1-P)\sum_{j}s_{j}^{f}}\\ & \;+(1-s)\left[P+(1-P)\frac{P(1-\sum_{j}s_{j}^{f})}{P+(1-P)\sum_{j}s_{j}^{f}}\right]. \end{array}$$

Equation (4.6) can be written in a simplified form. The reason we write it in this way is to show that the final people with the media opinion are composed of three parts, which correspond to the three terms of the right-hand side of equation (4.6): committed people, the amplification effect of networks on the opinions of committed people and the external influence of media. Further we find that the third term is composed of two parts, one is from the media effect when there are only people but no social influence between people, and the other is from the influence of mass media on people’s opinions through social connections. In other words, mass media can influence people’s opinions not only directly by global broadcasting but also indirectly by social interactions among people. The indirect influence of mass media on people’s opinions through interpersonal communication has been observed in empirical researches, such as the impact of a popular radio station on Rwandan genocide [68] and advertising’s effect on adolescents’ materialistic values [69]. From equations (4.3) and (4.6), we find that the final proportion of each opinion is related only to the media strength and the initial distribution of committed people. Ultimately, only the media opinion and the opinion(s) held by the committed people can survive.

**Figure 3.**
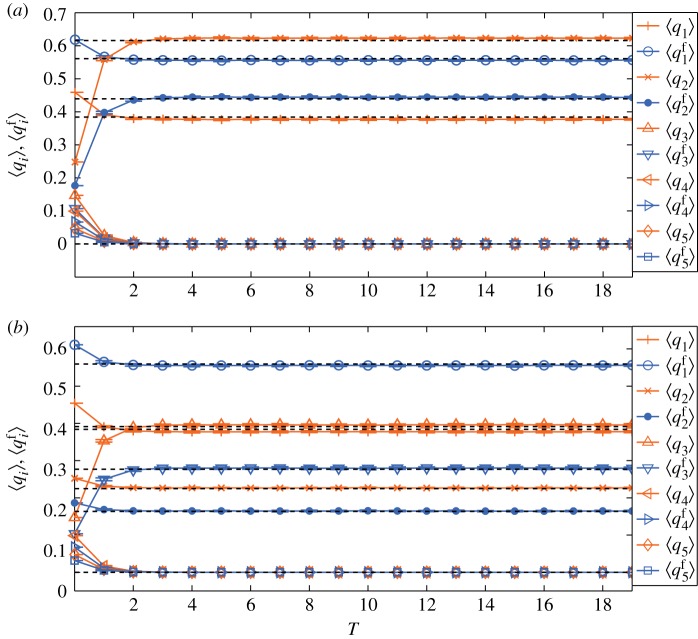
Evolutions of 〈*q*_*i*_〉 and $\left\langle q_{i}^{f}\right\rangle$ when *f*_*k*_∝*k*^0.5^. The unit of time *T* is *N*/(1−*s*) steps. The dashed lines indicate $\lim_{t\to \infty }q_{i}^{f}(t)$ and $\lim_{t\to \infty }q_{i}(t)$ obtained from equations (4.2), (4.3), (4.5) and (4.6). Error bars with ±1 standard deviation are shown. (*a*) *m*=2 and (*b*) *m*=3. At steady state both 〈*q*_*i*_〉 and $\left\langle q_{i}^{f}\right\rangle$ achieve good agreement with theoretical predictions. For both cases, the media opinion eventually wins.

We perform numerical simulations on the BA network of *N*=10^4^ and 〈*k*〉=6. We suppose that the initial network settings are the same as those in figure 2 and *P*=0.7. When *f*_*k*_∝*k*^0.5^, the corresponding numerical simulation results for *m*=2 and *m*=3 are shown in figure 3*a*,*b*, respectively. We find that the analytical predictions are still in good agreement with the numerical simulation results, and mass media outlets have significant influence on final opinion distribution.

Mass media makes both 〈*q*_*m*_〉 and $\left\langle q_{m}^{f}\right\rangle$ increase with time while 〈*q*_*i*_〉 and $\left\langle q_{i}^{f}\right\rangle$ (*i*≠*m*) decrease with time. In figure 3*a* although initially compared with *q*_1_ and $q_{1}^{f}$, both *q*_2_ and $q_{2}^{f}$ are smaller; at steady state *q*_2_ is larger than *q*_1_ and opinion 2 finally dominates the whole network due to the media effect. In figure 3*b*, media opinion 3 also ultimately wins although in the network there are no committed people holding the opinion.

**Figure 4.**
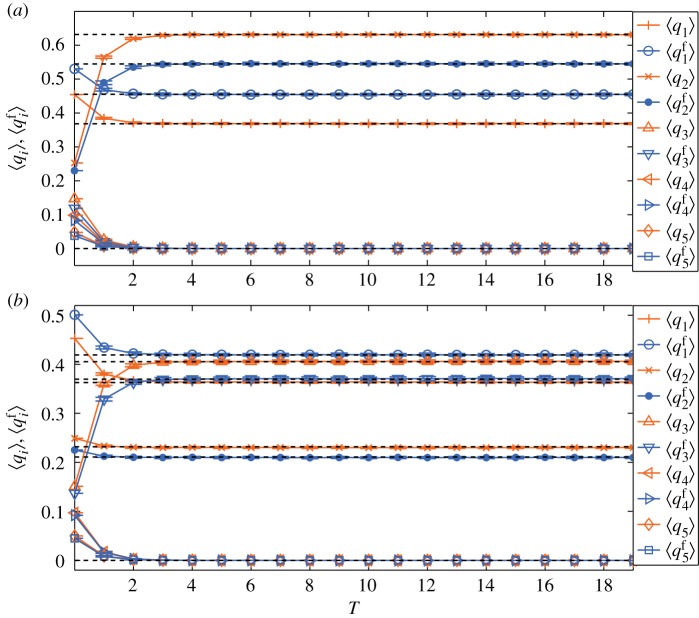
Evolutions of 〈*q*_*i*_〉 and $\left\langle q_{i}^{f}\right\rangle$ when *f*_*k*_ follows a uniform distribution in (0, 1). The unit of time *T* is *N*/(1−*s*) steps. The dashed lines indicate $\lim_{t\to \infty }q_{i}^{f}(t)$ and $\lim_{t\to \infty }q_{i}(t)$ obtained from equations (4.2), (4.3), (4.5) and (4.6). Error bars with ±1 standard deviation are shown. (*a*) *m*=2; (*b*) *m*=3. Similarly, for both cases, both 〈*q*_*i*_〉 and $\left\langle q_{i}^{f}\right\rangle$ at steady state achieve good agreement with analytical predictions, and the media opinion gradually wins.

When *f*_*k*_ follows a uniform distribution in (0, 1), the corresponding numerical simulation results for *m*=2 and *m*=3 are shown in figure 4*a*,*b*, respectively. The analytical predictions are also in good agreement with the numerical simulation results, and mass media outlets still have significant influence on the final opinion distribution. Similarly, media opinion eventually wins in the competing opinion diffusion. Because *f*_*k*_ reduces the impact of people with large degrees, compared with figure 3, in figure 4 at steady state $\left\langle q_{2}^{f}\rangle >\langle q_{1}^{f}\right\rangle$ when *m*=2 and $\left\langle q_{3}^{f}\rangle >\langle q_{1}\right\rangle$ when *m*=3.

We note that, for the degree-uncorrelated networks, at steady state 〈*q*_*i*_〉 and $\left\langle q_{i}^{f}\right\rangle$ accord well with the theoretical limits $q_{i}(\infty )$ and $q_{i}^{f}(\infty )$, respectively; thus given the initial conditions and using equations (4.3) and (4.6), we can present the competition between different opinions under the combined influence of social networks and mass media. From figures 3 and 4, we find that when media strength *P* is large enough, despite the initial disadvantage, media opinion will still win. While when *P* is small enough, another non-media opinion can win. Thus, there exists a ‘critical point’ for *P* which can be calculated from equations (4.3) and (4.6).

**Figure 5.**
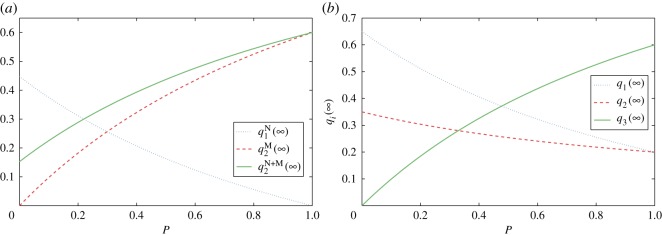
Evolutions of $q_{i}(\infty )$ with *P* when *f*_*k*_∝*k*^0.5^. (*a*) *m*=2 and (*b*) *m*=3. With the increase in media strength, the internal impact of social networks will weaken, whereas the external impact of media will strengthen.

Next, we will numerically illustrate the influence of media on opinion competition. Assume that the 100 largest degree people are the committed ones with opinion 1, but *s*_1_=*s*_2_=0.2 and *s*=0.4. When *m*=2, we use $q_{1}^{N}(\infty )$ to denote the amplification effect of networks on opinion 1, $q_{2}^{M}(\infty )$ to denote the external influence of media on opinion 2, and $q_{2}^{N+M}(\infty )$ to denote the combined influence of network and media on opinion 2. In figure 5, we present the evolutions of $q_{i}(\infty )$ with *P* when *f*_*k*_∝*k*^0.5^. From figure 5*a* we find that when *P* is increased from 0 to 1, the internal impact of the network for both opinions will be reduced to zero, while the influence of media on opinion 2 will increase to 1−*s*=0.6. When *P*=1, $q_{1}(\infty )=s_{1}=0.2$ and $q_{2}(\infty )=1-q_{1}(\infty )=q_{2}^{N+M}(\infty )+s_{2}=0.8$. According to equations (4.3) and (4.6), *P*≈0.22 is a critical point. When *P*<0.22 opinion 1 dominates, whereas when *P*>0.22 opinion 2 dominates. When *m*=3, the final proportion of each opinion is shown in figure 5*b*. When *P* increases from 0 to 1, both opinion 1 and 2 will decrease to *s*_1_=*s*_2_=0.2, whereas opinion 3 will increase to 1−*s*=0.6. When *P*<0.48 opinion 1 dominates, whereas when *P*>0.48 opinion 3 dominates.

**Figure 6.**
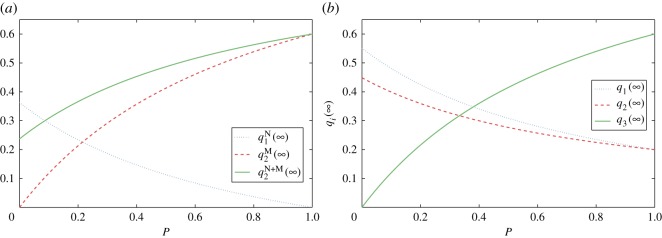
Evolutions of $q_{i}(\infty )$ with *P* when *f*_*k*_ follows a uniform distribution in (0,1). (*a*) *m*=2 and (*b*) *m*=3. Similarly, the increase in media intensity reduces the impact of networks, while increasing media impact.

When *f*_*k*_ follows a uniform distribution in (0,1), the evolutions of $q_{i}(\infty )$ with *P* are presented in figure 6 which is qualitatively like figure 5. Owing to the impact of people with large degrees on *f*_*k*_, when *P*=0, $q_{1}^{N}(\infty )$ and $q_{1}(\infty )$ in figure 5 are larger than those in figure 6. Thus, compared with figure 5, the critical point of *P* is smaller in figure 6: *P*≈0.09 in figure 6*a* and *P*≈0.38 in figure 6*b*.

## 5. Conclusion and discussion

In recent years, online social networks, such as Facebook, Twitter, Weibo (Chinese version of Twitter) and WeChat (Chinese instant messaging app), have not only experienced significant developments, but have also profoundly changed the way people communicate and access information [70,71]. The real-world and online social networks, as well as mass media, collectively influence and change people’s ideas, opinions and attitudes. Opinions on certain topics are competitive, such as brand selection for high-priced goods and candidate choice in political elections. In this paper, we propose an analysable model to study competing opinion diffusion on social networks. When there are no committed people, we get the probability that each opinion will eventually pervade the entire network. When there are committed people in networks, we concurrently consider the combined effects of social networks, individual attributes and media on opinion competition, and obtain the final proportion of each opinion at the steady state. The proportions are related only to media strength and the initial distribution of committed people in networks through which we can assess which opinion(s) will prevail.

The results in this paper are potentially valuable to agent-based modelling of biological and social systems where voter-like dynamics plays a decisive role, such as evolutionary games on networks [72–76]. The agent-based model studied in this paper is based on the voter model which is also known as the death–birth process in biology, and some other models such as birth–death process (Moran model or invasion process) and other evolutionary dynamics models in biology [58,59,77–84] can also be applied to the research on competitive opinion diffusion in social contexts. In competing diffusion, apart from degree-dependent persuasiveness, we can also assume that persuasiveness is related to opinion. In this case, each update would change the persuasiveness of an individual which can make the model analytically less tractable. Not all opinion competition models can be studied by analytical approaches. In many cases, it is difficult to get closed analytical solutions, and for some of the more complex models, it is probable that we will have to resort to more numerical methods, which demonstrates the practical limitation of approaches used in this paper. As pointed out earlier, opinion competition occurs not only on a single network, but also occurs simultaneously on multilayer networks. With the rise of social media software which can run on both PCs and mobile devices, massive data on human communication and social influence have been recorded; thus data-driven modelling has also attracted the attention of researchers. All of these give potential directions for further research.

## Data accessibility

The code supporting the paper is available at the Dryad Digital Repository (http://dx.doi.org/10.5061/dryad.h4b5d) [85].
